# Predicting the distribution of poorly-documented species, Northern black widow (*Latrodectus variolus*) and Black purse-web spider (*Sphodros niger*), using museum specimens and citizen science data

**DOI:** 10.1371/journal.pone.0201094

**Published:** 2018-08-08

**Authors:** Yifu Wang, Nicolas Casajus, Christopher Buddle, Dominique Berteaux, Maxim Larrivée

**Affiliations:** 1 Department of Natural Resource Sciences, McGill University, Montreal, Quebec, Canada; 2 Canada Research Chair on Northern Biodiversity, Université du Québec à Rimouski, Rimouski, Québec, Canada; 3 Insectarium de Montréal, Montréal, Québec, Canada; National Institute of Biology, SLOVENIA

## Abstract

Predicting species distributions requires substantial numbers of georeferenced occurrences and access to remotely sensed climate and land cover data. Reliable estimates of the distribution of most species are unavailable, either because digitized georeferenced distributional data are rare or not digitized. The emergence of online biodiversity information databases and citizen science platforms dramatically improves the amount of information available to establish current and historical distribution of lesser-documented species. We demonstrate how the combination of museum and online citizen science databases can be used to build reliable distribution maps for poorly documented species. To do so, we investigated the distribution and the potential range expansions of two north-eastern North American spider species (Arachnida: Araneae), the Northern black widow (*Latrodectus variolus*) and the Black purse-web spider (S*phodros niger*). Our results provide the first predictions of distribution for these two species. We also found that the Northern black widow has expanded north of its previously known range providing valuable information for public health education. For the Black purse-web spider, we identify potential habitats outside of its currently known range, thus providing a better understanding of the ecology of this poorly-documented species. We demonstrate that increasingly available online biodiversity databases are rapidly expanding biogeography research for conservation, ecology, and in specific cases, epidemiology, of lesser known taxa.

## Introduction

Data deficiency is the main obstacle for developing accurate distribution maps [1]. For most known species, museum specimens, private collections and historical literature are the major data sources to study species biogeography. Nevertheless, limited funding and the magnitude of the task to manually digitize museum specimen records result in few museums with digital, spatially-explicit databases, therefore limiting museum specimen data availability for the scientific community. Museums that have digital databases readily available sometimes lack information such as latitude, longitude, and precision of the GPS coordinates, which are necessary for generating species distribution models. Museum specimen data also often cover a limited time span [2, 3]. Observations or collections over the last 20 years are rarely found in museums, thus creating temporal gaps in database coverage. Such temporal gaps must be filled to produce reliable distribution predictions as recent anthropogenic climate and land use change hastens species range shifts [4, 5].

On the other hand, the internet allows convenient and fast data sharing, and this can help scientists rapidly gather research grade data with careful vetting. Currently, many citizen science (CS) initiatives not only provide natural history knowledge for the public, but also gather observation records of high quality [6]. These observations, in the form of collected specimens, photos, or sightings, are submitted to online databases by amateurs and researchers [7–13]. In platforms such as eBird and eButterfly, each observation submitted is vetted by regional experts who validate the precision of geographic coordinates, the observation date, and the species determination, sometimes correcting species identification when applicable. In other platforms such as iNaturalist and Bugguide, the observer community self-controls the quality of identifications (crowd-source identification) and possible misidentifications are questioned and moved to a special forum waiting for expert confirmation. Citizen Science platforms also often provide an open-access to all users. Many studies have been successful at using citizen science data in tracking species distribution, from birds to invasive plants [4–6, 14, 15]. Occurrence data originating from museum collections and citizen scientists are generally complimentary in their geographic coverage, which often improves the predictive power of species distribution models [7–12, 16]. Combining museum with citizen science data could be an optimal choice to predict and determine ranges and range shifts over time for under-documented species.

Determining reliable species distributions is a complex process requiring large amounts of data and effort which used to be only possible for well documented taxa. Species range shifts due to global change also adds complexity to the determination of species distribution [17–19]. Temperature and precipitation generally drive the distribution of temperate arthropods, particularly when modelling temperate spider distributions [20–25]. More precisely, the minimum temperature of the coldest period directly relates to the capacity of surviving through the winter forth Brown recluse spider (*Loxosceles reclusa*) [26]. Warming winter temperatures and extension of the growing season into the fall may delay such constraints and allow longer active periods through the delay in the first killing frosts increasing the possibility of northward range expansion [27–29]. Summer temperature regimes relate to reproductive success of some spider species, for example *Latrodectus hasseltii* [23]. Moisture level represented by precipitation data is also influential for terrestrial arthropod distribution as inadequate or excessive precipitation may lead to excessive stress due to extreme variations in moisture regimes [23, 24, 30].

The mapping complex effect from climate change on species distribution is simplified by major developments in species distribution model (SDM) algorithms and user-friendly interfaces over the last decade which allowed researchers and conservation practitioners to build reliable distribution models [31–33]. The ability of some SDMs to handle presence-only data also enables the determination of more detailed ranges of less-known taxa as these models can work with limited and often spatially-biased occurrence records, and even with data carrying some spatial uncertainty [34, 35]. Such improvements in the performance of SDMs coupled with the increasing availability of online research-grade distribution data greatly strengthen our capacity to predict the ranges of poorly documented taxa [16, 36].

Knowing the distribution of poorly documented taxa helps to understand their ecology as it provides information about where populations occur, along with their habitat requirements. This also potentially allows forecasting the species vulnerability to environmental change or human activities [37]. Determining potential habitat is also essential for planning species management strategies. Moreover, ecosystem management often uses the presence or absence of certain species as ecological indicators [38]. Poorly known species are sometimes assigned with inappropriate conservation status. New knowledge on their biogeography can prompt downgrading or upgrading of their conservation status [39]. In addition, some poorly known species are potential disease vectors or are venomous. Thus, knowing their range is crucial for people to apply corresponding management and public education plans.

We selected two poorly documented species of spiders: *Latrodectus variolus* (Walckenaer) (Northern black widow) and *Sphodros niger* (Hentz) (Black purse-web spider) to determine if reliable species distribution models can be made for poorly known species by combining museum and citizen science data. North black widow is a habitat generalist, it can be found from mesic to xeric deciduous forest and builds web high up in trees [40]. It also inhabits in human modified landscapes such as pine plantations and downed fence posts, building webs close to ground and in small burrows [41]. The black widow clade is known for the venomous bite of its species [42] and represents a human health concern throughout its range. Closely monitoring its distribution is thus important. The Black purse-web spider is cryptic, poorly known, and ranked as vulnerable in Virginia, US, yet its distribution is poorly understood as very few specimens have been collected [43]. *Sphodros niger* is also more habitat selective than many other spider species, preferring dry sandy/rocky woodland area [44–46]. Knowing more precisely its potential distribution would facilitate its management at regional levels by more reliably identifying its potential bioclimatic niches.

Another reason to choose these two species was due to the feasibility of species-level identification base on obvious features. Citizen science records are often collected by non-experts who do not know key features for species identification and only take photos of the whole animal. Obvious body features allow species-level identification to be feasible with such photos. More importantly, crowd-source identification may not be done by experts and misidentification is possible [47]. Thus, choosing species that can be accurately identified based on easily visible body features can simplify the process of re-validating crowd-source identification and insuring data accuracy.

The first objective of this research was to assess whether combining citizen science and museum data allows to successfully model the distribution of two poorly documented spider species. Our second objective was to determine the suitable bioclimatic niches of the two species and explore whether these species expanded north as recently documented for other taxa in North America [4, 48].

## Materials and methods

### Species data

We gathered distribution data from various sources including museums, research centers, literature, personal collections, and online citizen science projects [7, 8, 12]. Data collected from museums or institutes were accessed through open online database or through literature that cited these specimens (details see S1 and S2 Files), except records from Canadian National Collection which were collected through visiting this institute. To standardize the data collected online and to maximize validity of our dataset, we removed any records that could not be determined to species-level based on provided photos. *Latrodectus variolus* and *S*. *niger* can be both identified with a sufficiently high accurate rate by their unique body coloration and patterns. For *L*. *variolus*, the majority of both male and female have a disconnected hour-glass pattern on their ventral abdomen contrasting with the partially sympatric *L*. *mactans* to the south which mostly have complete hour-glass marks. Overall coloration of *L*. *variolus* also distinguish them from other *Latodectus* species and guide used for vetting can be found in McCrone and Levi [40], Jensen [49], and Wilson [41]. For *S*. *niger*, its big forward-projecting chelicerae distinguish it from spiders of other families. The overall black to dark reddish-brown coloration and stubby brown to black legs differentiate them from other Atypidae species (description see reference) [46, 50–52]. We also removed the ones that were suspected of being pet animals, for example photos taken in vivarium setting. When only locality information was available with no geographic coordinates, we calculated the geographic uncertainty of the given locations using Georeferencing Quick Reference Guide [53]. Records with uncertainty above 10 km were removed from analysis as their precision was beyond our grid cell size which is 10 km by 10 km. In total, 97 Black purse-web spider records and 164 Northern black widow records were used for modeling their distribution (Table 1).

**Table 1:** Summary of occurrence data available for *Sphodros niger* and *Latrodectus variolus* distribution models, including the sources of data, their period of collection, and sample sizes. Other sources include private collections, personal observations, and news articles.

Species	1960–1989 from museum, literature, and other sources	1990–2016 (1990–2015 for *S*. *niger*) from museum, literature, and other sources	1960–1989 from citizen sciences	1990–2016 (1990–2015 for *S*. *niger*) from citizen sciences	Total
*Sphodros niger*	44	39	0	14	97
*Latrodectus variolus*	22	47	0	95	164

### Environmental data

Climate data were constructed with ANUSPLIN, a regression splines interpolation, using all available weather station data in North America [54, 55]. Climate data resolution is at 10 arc minute resolution annually from 1960 to 2010 and was divided into two time-periods: 1960–1989 and 1990–2010. Then climate data of the two periods were averaged respectively using raster calculator in ArcGIS to represent historical and current climate. These climate data included: annual mean temperature, minimum temperature of the coldest period, mean temperature of the warmest quarter, mean temperature of the coldest quarter, annual precipitation, and precipitation seasonality (coefficient of variance).

### Species distribution models

Species distribution models were created using MaxEnt 3.3.3k [56, 57]. It is one of the best distribution model techniques using presence-only data [31, 34, 58]. MaxEnt is widely used to predict distribution of many taxon, including spider distribution [59]. MaxEnt requires two types of data for modeling distribution, appropriate environmental layers and species distribution data containing latitude and longitude.

We constructed the current distribution model using climate data between 1990 and 2010 and species occurrence data between 1990 and 2016 (1990 to 2015 for *S*. *niger*). It is common practice in species distribution modelling that observations obtained slightly outside of the environmental variable timeframe are included, especially when occurrence data are rare [16, 60]. The historical distribution model used both occurrence and climate data between 1960 and 1989. Background points are used in presence-only species distribution model like MaxEnt to model pseudo-absences for species. Background points (n = 10,000) were identified across the study area. Models were constructed using the “samples with data” approach, using 10-fold cross-validation of model outputs against held-back species observations and converted to binary predictions of presence/absence using a 0.39 threshold [61, 62]. The models were iterated 100 times and the mean of these model outputs was used as the consensus estimate of each species’ distribution across the modelling extent. The accuracy of our models was evaluated using the area under the receiver operating characteristic curve (AUC), correlation coefficients (COR), and true skill statistics (TSS) [34, 63–66]. Models with good predictive performance have an AUC value close to 1. COR show how well the predicted value fits real data [31]. High COR values indicate strong positive correlation between predictions and actual presences of the species, which proves good model performance with high confidence [31, 67]. TSS score between 0.40 and 0.75 shows good predictive performance of the model while TSS score above 0.75 shows excellent performance [64].

Since not all 10 km x 10 km grid cells were sampled for species presence in our study area, some spatial bias could affect model predictions [68–70]. Several methods can address this bias in species distribution models [35, 71, 72], and we followed the method used by Elith *et al*. [32], which up weights a grid cell with fewer neighbors in geographic space. We calculated the number of occurrences in a chosen neighborhood for each grid cell and weighted this number by a Gaussian function (see [71] for further details). The resulting bias grids showed higher weight in areas that were more intensively sampled. In the Gaussian function, the standard deviation parameter must be specific to the species. We followed the recommendations of Clements and Rayan [71] and defined this parameter as the diameter of a “circular” home range of the species. We then estimated the surface of the home range using the 95% kernel approach [73].

We tested the existence of potential range shifts in the two spider species by comparing the respective northern limit of modeled historical distribution and current distribution. A paired t-test was run to compare the difference between the latitudes of predicted northern limits during the two periods. We also calculated the mean latitudinal difference between the modeled northern limits to quantify the proposed range shifts. Average latitudinal differences and associated standard deviations were converted to distances (km) to facilitate result interpretation.

## Results

**Fig 1:**
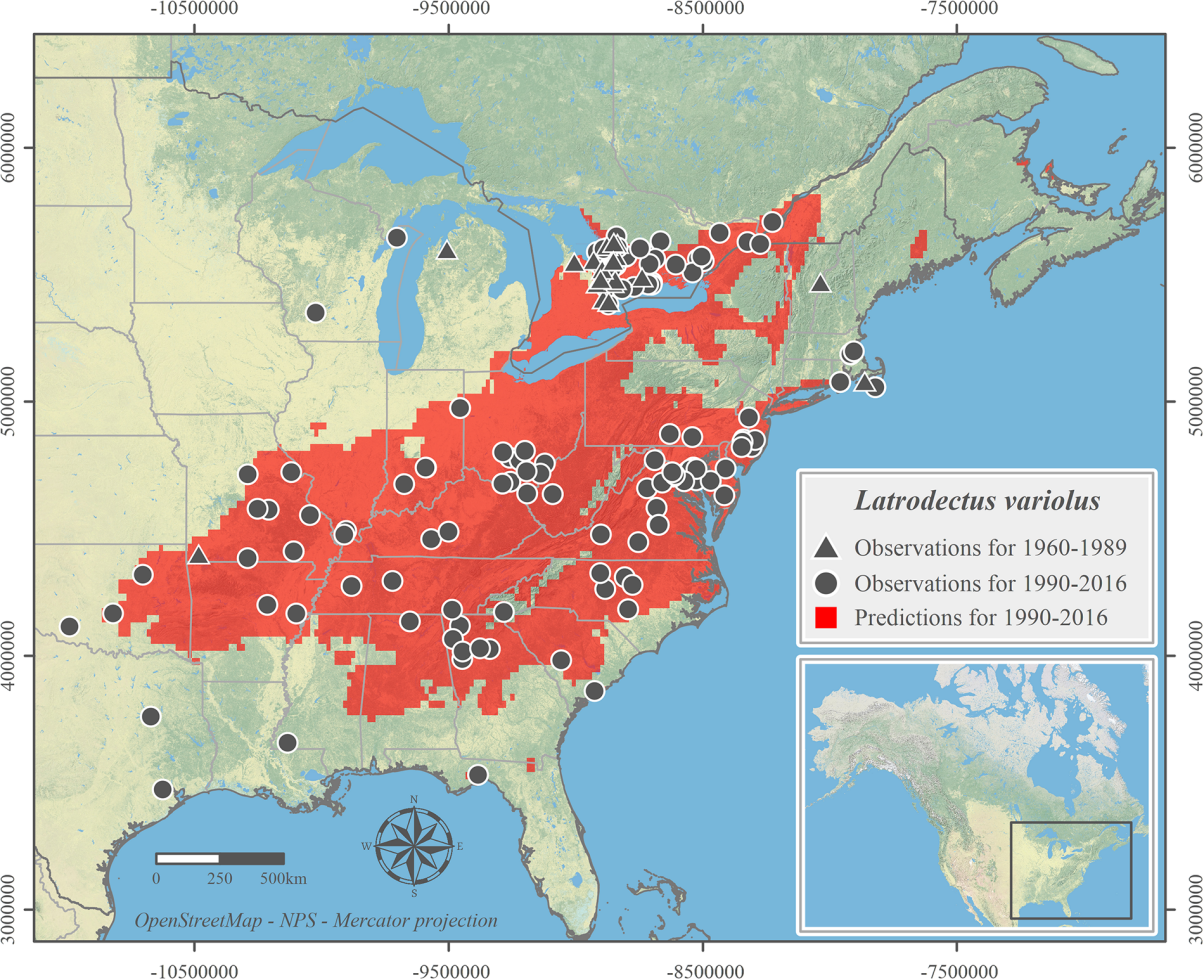
Suitable climatic habitat of *Latrodectus variolus* predicted from 1990–2016 observation records. Observation records are shown for both 1960–1989 and 1990–2016.

**Fig 2:**
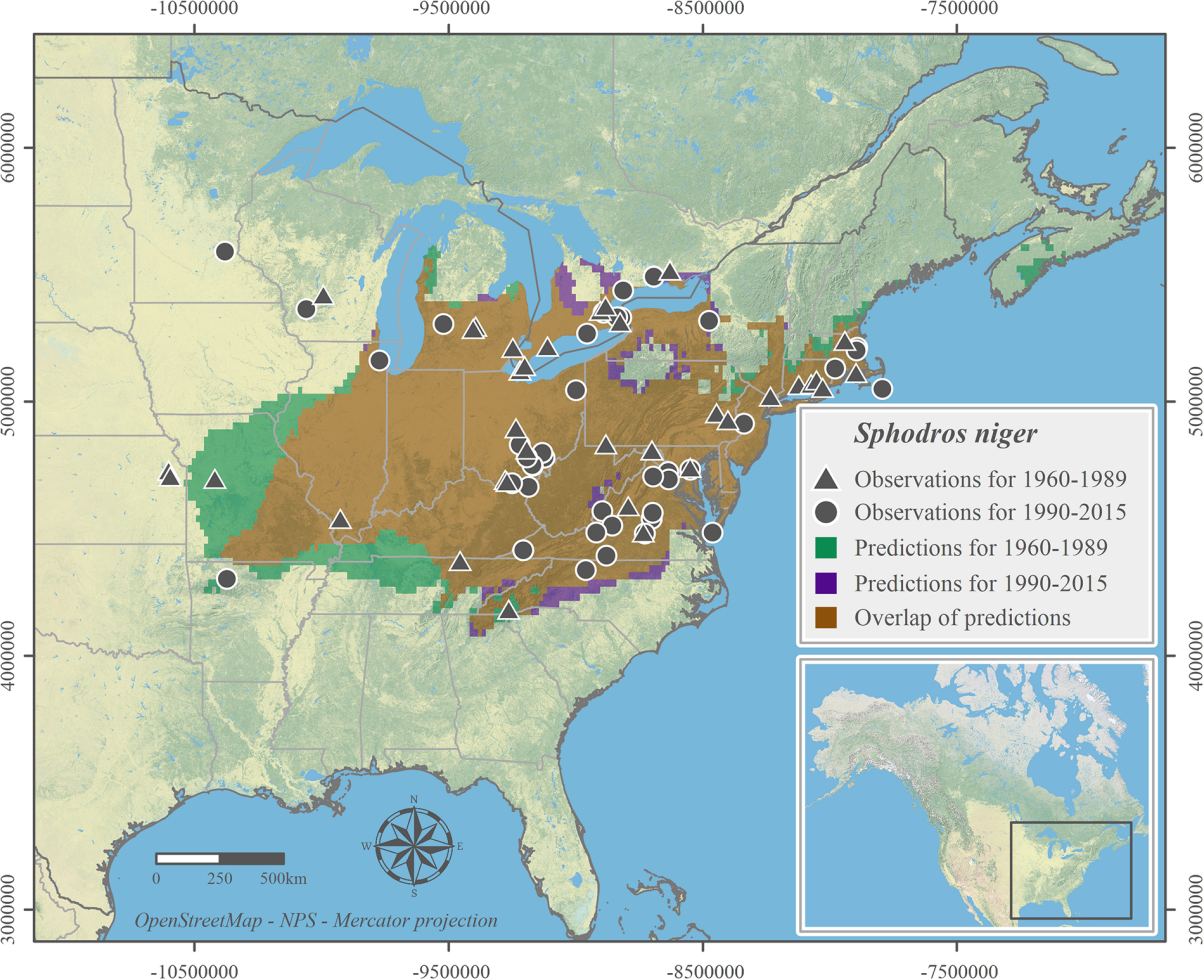
Suitable climatic habitat of *Sphodros niger* predicted from 1960–1989 and 1990–2015 observation records. Observation records are shown for both 1960–1989 and 1990–2015.

We successfully modeled the current distributions of *Latrodectus variolus* (Fig 1) and *Sphodros niger* (Fig 2). The historical distribution of *S*. *niger* was modeled successfully (Fig 2) but we failed to predict the historical distribution for *L*. *variolus* which is not included in maps. All illustrated range predictions had very high AUC, COR and TSS values (Table 2).

**Table 2:** Predictive performance of *Latrodectus variolus* and *Sphodros niger* distribution models assessed through the receiver operating characteristic curve (AUC), correlation coefficients (COR), and true skill statistics (TSS). Values are the average (± SD) of 100 iterated models.

Model	AUC	COR	TSS
***Latrodectus variolus***			
**1990–2016**	0.948 ± 0.009	0.384 ± 0.021	0.709 ± 0.086
***Sphodros niger***			
**1960–1989**	0.965 ± 0.013	0.277 ± 0.030	0.754 ± 0.161
**1990–2015**	0.967 ± 0.018	0.293 ± 0.029	0.802 ± 0.145

The result of the *S*. *niger* 1990–2015 model indicates new bioclimatic niches north of the modeled 1960–89 distribution range (especially in Ontario, Canada), but also a contraction in the southwest of the modeled distribution range, especially in Arkansas, Missouri and Tennessee. Two recent occurrences originating from CS in Minnesota and Wisconsin suggest a possible broader current range to the northwest for *S*. *niger* (Fig 2). Our northern edge shift calculation was only performed in *S*. *niger* models as the historical range model of *L*. *variolus* was not successful. The northern limit comparison reveals that there is a significant difference between the two northern edges of the models (n = 294, t = 5.751, df = 293, p-value < 0.001). The predicted northern shift as the mean difference between the two edges was -35 km with a high SD of ± 107 km.

The contribution of each environmental factor towards the final models was shown in the following table (Table 3). Mean temperature of the warmest quarter was the most important bioclimatic variable determining the current modeled range of *L*. *variolus* while mean temperature of the coldest quarter was the most important factor determining the modeled range of *S*. *niger* in both time periods (Table 3).

**Table 3:** The importance of the six bioclimatic predictors used in habitat range models of *Latrodectus variolus* and *Sphodros niger*.

Model	Mean annual temperature (%)	Minimum temperature of the coldest month (%)	Mean temperature of the warmest quarter (%)	Mean temperature of the coldest quarter (%)	Total annual precipitation in ml (%)	Precipitation seasonality (%)
***Latrodectus variolus***						
**1990–2010**	1.4 ± 0.9	1.3 ± 1.2	67.0 ± 2.9*	2.4 ± 0.8	4.9 ± 1.4	23.0 ± 3.0
***Sphodros niger***						
**1960–1989**	14.7 ± 3.5	12.2 ± 10.6	23.1 ± 3.7	41.1 ± 15.4*	3.0 ± 1.5	5.8 ± 2.3
**1990–2010**	0.2 ± 1.0	29.2 ± 14.9	14.5 ± 4.5	39.0 ± 15.1*	9.6 ± 3.9	7.6 ± 3.5

The percentages with their associated standard deviations are the contribution of each factor to the final results.

* Asterisk mark highlights the most influential factor for each model.

## Discussion

We provide the first bioclimatic range predictions for two poorly known spider species, *Latrodectus variolus* and *Sphodros niger*. Our results show that constructing reliable species distribution models (very high AUC, COR and TSS values) is feasible for poorly documented taxa using multi-sourced data including well validated and curated online citizen science data. Phenological and range-shift responses for species in North America occurred mostly after 1960 [18], as did directional climate change, which is very likely due to human activities [74]. Therefore, 1960 was used as a historical baseline in which to construct our species distribution models. 67% of our *L*. *variolus* post 1990 records come from vetted online CS data (Table 1), which emphasizes the importance of this new data source in increasing our understanding of the distribution and biology of poorly known taxa. The failure to model the historical distribution range (1960–1990) of *L*. *variolus* is due to limited data records, 22 occurrences in total, which is too close to the minimum sample size for modeling wide-spread species [75]. This data deficiency induced failure further reinforces the necessity of digitalizing all available historical records and the incorporation of CS data for studying poorly-documented species. We successfully overcame the challenges described by Dickinson *et al*. [76], particularly the geographical biases inherent to CS data. We incorporated observer errors and spatial sampling bias through multilayered data vetting and constructing sampling bias grid [32, 71]. Our method provides a valuable template for similar studies in future.

The distribution of both species was strongly driven by temperature. Mean temperature of the warmest quarter is the driving factor of the distribution of *L*. *variolus* (Table 3). Summer temperature influences spiders’ life history in multiple aspects including breeding behavior, web construction, prey availability, growth rate, and dispersal behavior [77–80]. For *S*. *niger*, mean temperature of the coldest quarter is the most influential environmental factor in both models. Temperature is closely related to the winter survival rate of many spider species and thus often defines their northern distribution limits [26].

We were not able to provide statistically significant evidence to support the proposed north shift scenario as either the historical range model failed (*L*. *variolus*), or the range shift calculation had a high SD (*S*. *niger*) which devalued the result. Nevertheless, our model predictions of the current northern limits of both species are based on very conservative model thresholds and both predicted ranges extend beyond previously documented regions (Figs 1 and 2) [43]. For *L*. *variolus*, we found that the northern most observation for the 1990–2016 period (located in Quebec) was 94 km north of the northern most observation for 1960–1989 (located in Ontario). The predicted suitable climatic niche of *L*. *variolus* for the 1990–2016 extends another 50 km north to specimens having yet to be recorded at the northeast of Montréal, QC. The model of *L*. *variolus* is also in accordance with recent observations north of its previously known range. These observations in Eastern Ontario [81, 82] and Southern Québec (from Montreal Insectarium entomological enquiry services in 2012, 2015 and 2016) provide strong empirical support to our northern range expansion hypothesis. The Montreal Insectarium has been answering on average 1500 public entomological inquiries since 1990 and received all three inquiries pertaining to *L*. *variolus* in Quebec after 2012. Thus, these observations beyond the historically known northern limit were not the result of the multiplication of CS projects in recent years. Climate change, which influences seasonal temperature pattern, could be a strong contributor to the increase in occurrences of Northern black widow beyond their historical northern limit [17, 23, 83, 84]. Similar expansion pattern has been revealed in other arthropod species in the northeast of North America where our focal species ranges are located, for example the giant swallowtail butterfly (*Papilio cresphontes*) [85] and other conspicuous butterfly species [4, 48]. Another spider of health concern in North America, the Brown recluse (*Loxosceles reclusa*), was shown to potentially expand their distribution range northwards under future climate change scenarios [84]. Thus, these two spider species are also possible to respond similarly to the climate change induced relaxation of limiting factors and have expanded northwards.

Quick and successful colonization to new habitat is particularly possible for *L*. *variolus* as this species is a habitat and prey generalist. Thus, it is more likely to survive in new environments after long-distance ballooning, the main dispersal method of spiders [78, 86–88]. *L*. *variolus* also has a higher metabolic rate compared to other theridiid species, allowing it to have larger clutch sizes and thus a higher reproduction rate [89]. Therefore, *L*. *variolus* has several of the key assets generally associated with good colonizers. For *S*. *niger* being a habitat specialist, colonizing new habitat beyond its currently known range extent might be slower and less efficient due to the random aspect of long distance ballooning.

Different requirement on habitat type may also be the explanation to the different most important environmental factor in predicting models (Table 3). *Sphodros niger* being a habitat specialist preferring dry sandy/rocky woodland area, escaping cold winter might be difficult while *L*. *variolus* can be found frequently in man-made shelters. As the result, mean temperature of the coldest quarter is found to be the most influential factor for mapping *S*. *niger* distribution. On the other hand, relatively higher metabolic rate of *L*. *variolus* comparing to other theridiid species might be the reason why mean temperature of the warmest quarter was the most important bioclimatic variable determining its current range. High metabolic rate is a trait evolved to adapt to cold environment and thus cold temperature may not be a critical constrain to *L*. *variolus* comparing to the heat in summer [90].

Our models show the first reliable distribution maps of these two species and both species have potential distribution ranges beyond currently documented regions [43]. The logical next step is to conduct sampling efforts in typical habitats associated with these species in our predicted range to further validate the models. However, detection of range expansion of low density cryptic species such as *L*. *variolus* and *S*. *niger* would be the equivalent of searching for a needle in a hay stack for a small group of experts across such a wide region. Thus, we propose to call on citizen scientists by launching a monitoring project through a platform such as Bugguide and iNaturalist to produce a large-scale sampling effort. This would represent a rapid, low cost, highly efficient, and innovative way to test these large scale predictive models. Although the risk of being bitten by northern black-widows is low, such a species monitoring approach mobilizing public will include safety guidelines to prevent health issues from the participants.

On the other hand, local health authorities should be informed of the documented presence of *L*. *variolus* north of its previously known range to have appropriate material and response protocols in case any citizen is bitten by this species, known to cohabit with humans in and around their buildings. For *S*. *niger*, new information about its potential range can serve as important guidelines to authorities and stakeholders working on conservation efforts for this species at various governmental levels where it is at risk.

Our distribution models not only increase our understanding of the current distribution of these two poorly documented spider species, they also provide guidance for corresponding public health and conservation management strategies. More importantly, we must emphasize that data collected from citizen science initiatives and other online scientific open-sources, with the incorporation of observer errors and spatial sampling bias parameters, enabled us to produce these reliable distribution models. Even if online information needs careful vetting before use, we show that citizen science initiatives can provide valuable occurrence data even for under-sampled rare species and habitat specialists. Open-access and digitalized data from museums and CS platforms will likely become an important and convenient data source for natural science research.

## Supporting information

S1 File *Latrodectus variolus* record. This file contains occurrence records of *Latrodectus variolus* collected and used in this research.
(XLS)

S2 File *Sphodros niger* record. This file contains occurrence records of *Sphodros niger* collected and used in this research.
(XLS)
